# Crystal structure of [3,10-bis­(4-fluoro­pheneth­yl)-1,3,5,8,10,12-hexa­aza­cyclo­tetra­deca­ne]nickel(II) diperchlorate

**DOI:** 10.1107/S2056989020016795

**Published:** 2021-01-22

**Authors:** Chee-Hun Kwak, Mee Chang

**Affiliations:** a Department of Chemistry Education, Sunchon National University, 255 Jungang-ro, Sunchon, 57922, South Korea; b Polymerization Manufacturing Technology Research Team, Lotte Chemicals, 334-27 Yeosu Sandan-ro, Yeosu, 59616, South Korea

**Keywords:** crystal structure, whole-mol­ecule disorder, 3,10-bis­(4-fluoro­pheneth­yl)-1,3,5,8,10,12-hexa­aza­cyclo­tetra­deca­ne, 4-fluoro­phenethyl side chain, nickel(II) complex, *trans*-III configuration

## Abstract

The square-planar nickel(II) title complex, [Ni(C_24_H_36_F_2_N_6_)](ClO_4_)_2_ or [Ni*L*](ClO_4_)_2_ (*L* = 3,10-bis­(4-fluoro­pheneth­yl)-1,3,5,8,10,12-hexa­aza­cyclo­tetra­deca­ne) was synthesized by a one-pot reaction of template condensation and its X-ray crystal structure was determined. The nickel(II) ion lies close by a twofold axis and the complex displays whole-mol­ecule disorder. Ligand *L*, a hexa­aza­cyclo­tetra­decane ring having 4-fluoro­phenethyl side chains attached to uncoordinated nitro­gen atoms, adopts a *trans* III (*R,R,S*,*S)* configuration. The average Ni—N bond distance is 1.934 (9) Å, which is quite similar to those of other nickel(II) complexes with similar ligands. The nickel(II) ion is located 0.051 (7) Å above the least-squares plane through the four coordinated N atoms. The average C—N bond distance and C—N—C angle involving uncoordinated nitro­gen atoms are 1.425 (12) Å and 118.0 (9)°, respectively, indicating a significant contribution of *sp* ^2^ hybridization for these N atoms. The inter­molecular N—H⋯O, C—H⋯O/F hydrogen bonds of the complex form a network structure, which looks like a seamless floral lace pattern.

## Abstract 2

A new nickel(II) complex of a hexa­aza­macrocycle containing 4-flurophenethyl pendant arms was synthesized by metal template condensation in a one-pot reaction of formaldehyde and amines in the presence of nickel(II) ions and its X-ray crystal structure was determined.

## Chemical context   

A metal template condensation reaction with formaldehyde and appropriate amines is a useful method for the synthesis of saturated polyaza­macrocyclic complexes. It often produces new macrocyclic complexes in one-pot reactions with high yield *via* selective routes (Salavati-Niasari & Davar, 2006 ▸; Salavati-Niasari & Najafian, 2003 ▸; Suh, 1996 ▸). The introduction of pendant arms into polyaza­macrocyclic ligands has, sometimes, changed the structural and chemical properties of the complexes considerably (Hermann *et al.*, 2008 ▸; Jee *et al.*, 2003 ▸; Alexander, 1995 ▸; Kang *et al.*, 1995 ▸). The information derived from polyaza­macrocyclic complexes containing pendant arms helps in the understanding of apical effects in the biological behavior of tetra­aza­macrocyclic metalloenzymes having a square-planar geometry (Liang & Sadler, 2004 ▸; Costamagna *et al.*, 2000 ▸). Furthermore, the donor atoms in the pendant arms of these macrocyclic complexes can be coordinated to another metal ion or participate in hydrogen bonding. Consequently, these complexes can be applied in the field of supra­molecular chemistry or metal–organic frameworks. In the nickel(II) complex 8-(pyridin-4-ylmeth­yl)-1,3,6,8,10,13,15-hepta­aza­tri­cyclo[13.1.1.1^13,15^]octa­decane, inter­molecular hydrogen bonding between the nitro­gen of the pendant pyridine and coordinated water produces a one-dimensional chain structure (Jee *et al.*, 2003 ▸). In particular, many supra­molecular studies including metal–organic frameworks using complexes of 3,10-bis­(alk­yl)-1,3,5,8,10,12-hexa­aza­cyclo­tetra­decane-type ligands are available because they can be obtained by easy synthetic routes using metal template reactions (Min & Suh, 2001 ▸; Kang *et al.*, 1999 ▸; Suh *et al.*, 1994 ▸). The nickel(II) complex of 3,10-bis­(2-cyano­eth­yl)-1,3,5,8,10,12-hexa­aza­cyclo­tetra­decane produces a coordination polymer with each nickel(II) ion in the macrocycle units coordinating to two nitrile pendant groups of neighboring macrocycles (Suh *et al.*, 1994 ▸). In the nickel(II) complex of 3,10-bis­(pyridin-4-ylmeth­yl)-1,3,5,8,10,12-hexa­aza­cyclo­tetra­decane, hydrogen-bonding inter­actions between nitro­gen atoms in pendant pyridine rings, structural water mol­ecules and hydrogen atoms of the secondary amine of the macrocycle link the macrocyclic complexes, resulting in a two-dimensional network (Min & Suh, 2001 ▸). In addition, many studies on metal–organic frameworks have been performed using complexes of 3,10-bis­(alk­yl)-1,3,5,8,10,12-hexa­aza­cyclo­tetra­decane-type ligands (Jeoung *et al.*, 2019 ▸; Stackhouse & Ma, 2018 ▸; Lee & Moon, 2018 ▸; Lin *et al.*, 2014 ▸). In this communication, we report the preparation of a new nickel(II) complex [Ni*L*](ClO_4_)_2_, where *L* is a 3,10-bis­(alk­yl)-1,3,5,8,10,12-hexa­aza­cyclo­tetra­decane ligand having 4-fluoro­phenethyl pendant arms at positions 3 and 10, and its structural characterization by single-crystal X-ray crystallography.
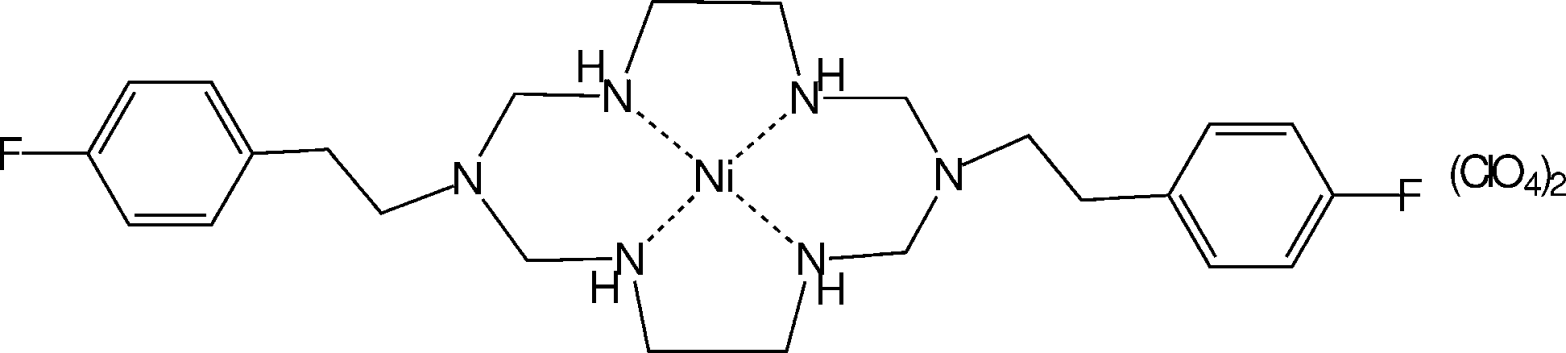


## Structural commentary   

**Figure 1:**
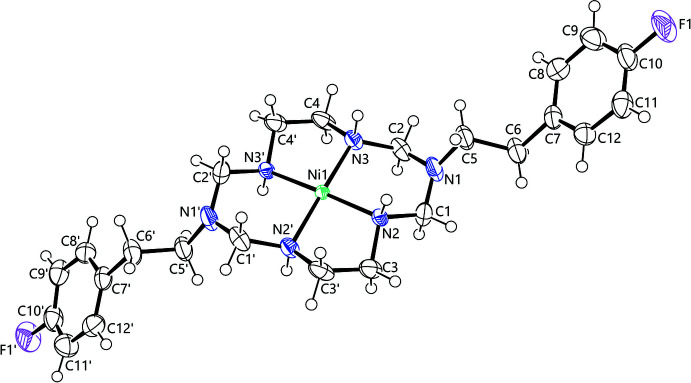
Mol­ecular structure of one of the whole-mol­ecule disorder component mol­ecules of [Ni*L*]^2+^ with displacement ellipsoids at 50% probability level. The second disorder component, generated by (1 − *x*, *y*, 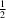 − *z*) is omitted for clarity.

The mol­ecular structure of the title compound is shown in Fig. 1 ▸. Both the complex and perchlorate anion display disorder. The Ni^II^ ion lies close by a special position (twofold axis) and the [Ni*L*]^2+^ complex occurs in two orientations with fixed occupancies of 0.50. The refinement of this whole-mol­ecule disorder needed additional restraints (see *Refinement* section). The occupancies of the disordered perchlorate ion are 0.795 (7) and 0.205 (7). The nickel(II) ion is coordinated to the four nitro­gens N2, N3, N2′ and N3′, and the complex has a square-planar coordination geometry. The 14-membered ring skeleton adopts the thermodynamically most stable *trans*-III configuration with *R,R,S,S* chirality of the four coordinated nitro­gen atoms (Barefield, 2010 ▸). The ligand *L* of the complex has two 4-fluoro­phenethyl pendant arms attached to the two uncoordinated nitro­gens (N1 and N1′) of the 14-membered 1,3,5,8,10,12-hexa­aza­cyclo­tetra­decane ring skeleton. The 4-fluoro­phenethyl pendants are positioned above and below the square coordination plane. The six-membered chelate rings adopt a chair conformation and the five-membered chelate rings assume a *gauche* conformation.

Selected bond distances and angles are listed in Table 1 ▸. The average Ni—N bond distance of 1.934 (9) Å is quite similar to those in square-planar nickel(II) complexes of various other related 14-membered polyaza macrocycles (Kang *et al.*, 1999 ▸; Suh *et al.*, 1998 ▸; Suh *et al.*, 1996 ▸). The bite angles of five-membered chelates are 86.5 (2)° for N2—Ni1—N2′ and 86.6 (3)° for N3—Ni1—N3′, respectively and those of six-membered chelates are 93.7 (4)° for N2—Ni1—N3 and 93.0 (4)° for N2′—Ni1—N3′, respectively. The four coordinating nitro­gen atoms (N2, N3, N2′ and N3′) are almost co-planar (r.m.s. deviation 0.010 Å). The nickel(II) ion is located 0.051 (7) Å above this least-squares plane showing a slightly square-pyramidal distortion. The N—C bond distances involv­ing the uncoordinated bridgehead nitro­gens (N1 and N1′) range from 1.398 (11) Å (N1—C1) to 1.481 (10) Å (N1′—C5′) and the average N—C bond distance is 1.425 (12) Å, which is significantly shorter than the other N—C single bond distances. Furthermore, the C—N—C bond angles involving these bridgehead nitro­gens range from 115.5 (7)° (C1—N1—C2) to 120.1 (8)° (C1′—N1′—C5′) and the average bond angle is 118.0 (9)°, which is distinctly larger than the ideal tetra­hedral angle. These results indicate a significant contribution of *sp* ^2^ hybridization of the bridgehead nitro­gen atoms (N1 and N1′) (Min & Suh, 2001 ▸; Kang *et al.*, 1999 ▸).

## Supra­molecular features   

**Figure 2:**
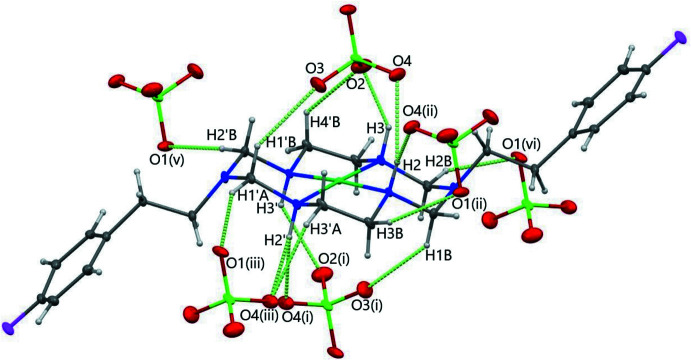
Hydrogen-bonding inter­actions involving the perchlorate anions in the crystal packing [Ni*L*](ClO_4_)_2_. Light-green dashed lines indicate N—H⋯O and C—H⋯O hydrogen-bonding inter­actions. Symmetry codes: (i) 1 − *x*, *y*, 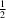 − *z*; (ii) 1 − *x*, 1 − *y*, 1 − *z*; (iii) *x*, −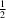 + *y*, 1 − *z*; (v) 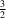 − *x*, 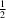 − *y*, 1 − *z*; (vi) −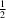 + *x*, 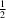 − *y*, −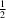 + *z*. Only one of the whole-mol­ecule disorder [Ni*L*]^2+^ components and the major component of the perchlorate anion are shown.

**Figure 3:**
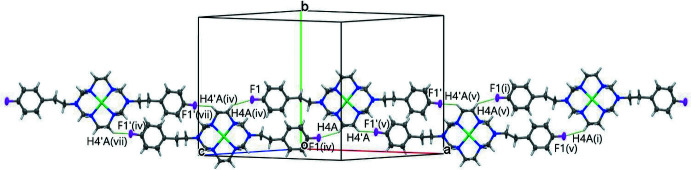
A view showing the one-dimensional chain propagation of rings formed by the inter­molecular hydrogen bonding between F1⋯ H4*A* and F1′⋯H4′*A* in [Ni*L*]^2+^. Symmetry codes: (i) 1 − *x*, *y*, 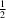 − *z*; (iv) 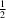 − *x*, 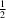 − *y*, 1 − *z*; (v) 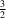 − *x*, 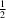 − *y*, −*z*; (vii) −1 + *x*, *y*, 1 + *z*. Only one of the whole-mol­ecule disorder [Ni*L*]^2+^ components is shown.

**Figure 4:**
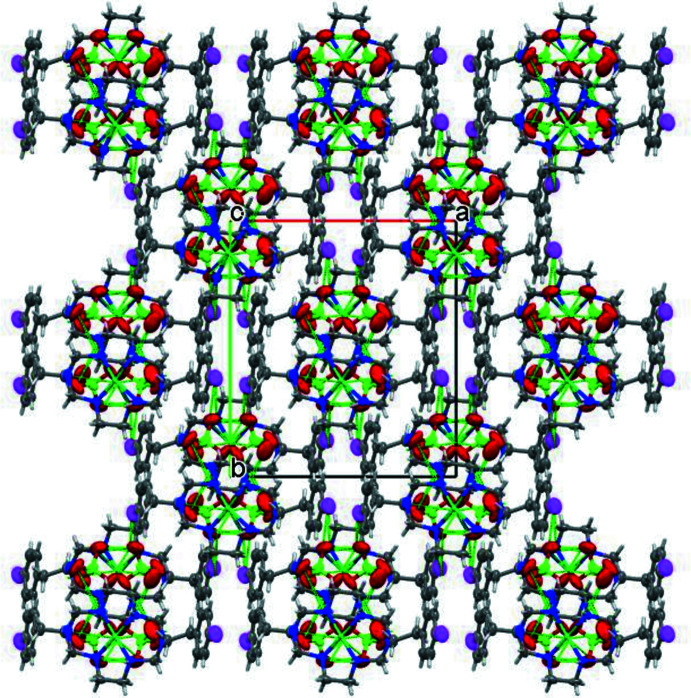
A view of the crystal packing of [Ni*L*](ClO_4_)_2_, which resembles a seamless floral lace pattern. Light-green dashed lines indicate hydrogen-bonding inter­actions.

There are several N—H⋯*A* (*A* = O) as well as C—H⋯*A* (*A* = O or F) hydrogen bonds in the crystal packing of [Ni*L*](ClO_4_)_2_. Hydrogen-bonding inter­actions between N—H or C—H groups of the ligand *L* and perchlorate oxygen atoms are summarized in Table 2 ▸ and illustrated in Fig. 2 ▸. In addition, fluorine atom F1 in one of the pendant phenyl groups of the macrocycle is involved in an inter­molecular inter­action with hydrogen H4*A* of a neighboring mol­ecule (Table 2 ▸ and Fig. 3 ▸). The other fluorine atom, F1′, takes part in a weaker hydrogen-bonding inter­action with H4′*A* of a neighboring mol­ecule [H4*A*⋯F1′ = 2.62 Å, C4′⋯F1′ = 3.312 (17) Å and C4′—H4′*A*⋯F1′ = 128.4 (8)°]. These inter­actions form a chain structure extending in the [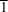
01] direction (Fig. 3 ▸). All of these inter­molecular hydrogen-bonding inter­actions lead to a network structure resembling a seamless floral lace pattern (Fig. 4 ▸).

## Database survey   

An Access Structures search of the Cambridge Structural Database (CSD, *via* CCDC Access Structures, December 2020; Groom *et al.*, 2016 ▸) resulted in 97 structures of complexes of 3,10-bis­(alk­yl)-1,3,5,8,10,12-hexa­aza­cyclo­tetra­decane derivatives and 13 structures of complexes of 1,8-bis­(alk­yl)-1,3,6,8,10,13-hexa­aza­cyclo­tetra­decane (different systematic name of the ligand). However, no results were found for the 3,10-bis­(4-fluoro­pheneth­yl)-1,3,5,8,10,12-hexa­aza­cyclo­tetra­decane structure.

In addition, 92 structures containing the 1,3,5,8,10,12-hexa­aza­cyclo­tetra­decane skeleton were found during a SciFinder search, but again no results were found containing the title complex. Most are classified as octa­hedral complexes, while only a few cases are square-planar nickel(II) complexes. The Ni—N bond distances are 1.931 (2)–1.934 (3) Å in the nickel(II) complex of 3,10-bis­(2-amino­eth­yl)-1,3,5,8,10,12-hexa­aza­cyclo­tetra­decane (Kang *et al.*, 1999 ▸), 1.934 Å in the nickel(II) complex of 3,10-dibenzyl-1,3,5,8,10,12-hexa­aza­cyclo­tetra­decane (Min & Suh, 2001 ▸), and 1.933 (3)–1.936 (3) Å in 1,8-dimethyl-1,3,6,8,10,13-hexa­aza­cyclo­tetra­decane (Benkada *et al.*, 2020 ▸), similar to those of the square-planar nickel(II) complexes of various other related 14-membered polyaza macrocycles. The Ni—N distances of 1.933 (4)–1.944 (4) Å in the nickel(II) complex of 1,8-dipentyl-1,3,6,8,10,13-hexa­aza­cyclo­tetra­decane (Park *et al.*, 2015 ▸) and the average Ni—N bond distance of 1.941 (6) Å in the nickel(II) complex of 3,10-bis­(α-methyl­naphth­yl)-1,3,5,8,10,12-hexa­aza­cyclo­tetra­decane (Min *et al.*, 2013 ▸) are a little longer than those of analogous complexes. However, the Ni—N distances of 1.927 (4)–1.932 (4) Å in the nickel(II) complex of 3,10-bis­(2-thio­phene­meth­yl)-1,3,5,8,10,12-hexa­aza­cyclo­tetra­decane (Su *et al.*, 2007 ▸) and 1.926 (1)–1.928 (1) Å in that of 3,10-bis­(2-hy­droxy­eth­yl)-1,3,5,8,10,12-hexa­aza­cyclo­tetra­decane (Kim *et al.*, 2002 ▸) are somewhat shorter than those of analogous complexes. In all these nickel(II) complexes of 3,10-bis­(alk­yl)-1,3,5,8,10,12-hexa­aza­cyclo­tetra­decane analogues, the nickel(II) ion is situated on an inversion center, except for the nickel(II) complex of 3,10-bis­(α-methyl­naphth­yl)-1,3,5,8,10,12-hexa­aza­cyclo­tetra­decane, which does not have an inversion center due to the chiral pendants of the macrocyclic ligand (Min *et al.*, 2013 ▸). The nickel (II) ion is exactly in the least-squares plane through the four coordinating nitro­gen atoms.

## Synthesis and crystallization   

A well-known one-pot reaction of template condensation was used for the preparation of the title complex (Salavati-Niasari & Rezai-Adaryani, 2004 ▸; Min & Suh, 2001 ▸; Kang *et al.*, 1999 ▸). 98% Ethyl­enedi­amine (1.1 ml, 16mmol), 99% 4-fluoro­phenethyl­amine (2.1 ml, 16 mmol), and 95% paraformaldehyde (1.44 g, 48 mmol) were slowly added to a stirred solution of 98% nickel(II) acetate tetra­hydrate (2.0 g, 8.0 mmol) in 50 ml of methanol. The solution was heated under reflux for 24 h and then cooled to room temperature. The solution was filtered, concentrated HClO_4_ was added to the filtrate, adjusting pH of the solution to 4, and it was kept in a refrigerator until a yellow-colored precipitate was formed. The product was filtered, washed with methanol, and dried in air. Single crystals for X-ray crystallography were obtained by recrystallization from hot water.

## Refinement   

Crystal data, data collection and structure refinement details are summarized in Table 3 ▸. H atoms were positioned geometrically and allowed to ride on their respective parent atoms [C—H = 0.93 Å (CH, aromatic), 0.97 Å (CH_2_) and N—H = 0.98 Å (NH_2_), and *U*_iso_(H) = 1.2*U*_eq_(C) or *U*_iso_(H) = 1.2*U*_eq_(N)].

The refinement of the whole-mol­ecule disorder employed the following constraints and restraints in *SHELXL*: (1) occupancy factors were set at 0.50, (2) the two chemically equivalent halves of the complex were restrained to be similar using the ‘SAME’ command, (3) the fluorinated benzene rings were given a weak ‘FLAT’ restraint, (4) Ni1 required a strong ‘ISOR’ restraint and (5) displacement factors for atom pairs related about the special position were constrained to be equal (EADP).

The perchlorate anion is disordered over two sets of atomic sites with occupancy ratios of 0.795 (7):0.205 (7).

## Supplementary Material

Crystal structure: contains datablock(s) I. DOI: 10.1107/S2056989020016795/vm2242sup1.cif

Structure factors: contains datablock(s) I. DOI: 10.1107/S2056989020016795/vm2242Isup2.hkl

CCDC reference: 2053166

Additional supporting information:  crystallographic information; 3D view; checkCIF report

## Figures and Tables

**Table 1:** Selected geometric parameters (Å, °)

Ni1—N3	1.925 (7)	N1—C2	1.401 (11)
Ni1—N2	1.933 (9)	N1—C5	1.469 (10)
Ni1—N3′	1.934 (7)	N1′—C1′	1.400 (11)
Ni1—N2′	1.943 (9)	N1′—C2′	1.408 (12)
N1—C1	1.398 (11)	N1′—C5′	1.481 (10)
			
N2—Ni1—N3	93.7 (4)	C1—N1—C5	118.9 (9)
N3—Ni1—N3′	86.6 (3)	C2—N1—C5	119.1 (8)
N2—Ni1—N3′	176.4 (4)	C1′—N1′—C2′	115.6 (7)
N2—Ni1—N2′	86.5 (2)	C1′—N1′—C5′	120.1 (8)
C1—N1—C2	115.5 (7)	C2′—N1′—C5′	118.5 (9)

**Table 2:** Hydrogen-bond geometry (Å, °)

*D*—H⋯*A*	*D*—H	H⋯*A*	*D*⋯*A*	*D*—H⋯*A*
N2—H2⋯O4	0.98	2.51	3.355 (17)	144
N2—H2⋯O4^i^	0.98	2.44	3.260 (16)	141
N2′—H2′⋯O4^ii^	0.98	2.43	3.332 (18)	152
N2′—H2′⋯O4^iii^	0.98	2.36	3.089 (17)	131
N3—H3⋯O2	0.98	1.97	2.819 (11)	144
N3′—H3′⋯O2^ii^	0.98	2.40	3.186 (11)	137
C1′—H1′*A*⋯O1^iii^	0.97	2.31	3.198 (13)	151
C1—H1*B*⋯O3^ii^	0.97	2.35	3.156 (16)	140
C1′—H1′*B*⋯O3	0.97	2.56	3.309 (15)	134
C2—H2*B*⋯O1^iv^	0.97	2.50	3.394 (16)	154
C3—H3*B*⋯O1^i^	0.97	2.48	3.35 (2)	149
C2′—H2′*B*⋯O1^v^	0.97	2.58	3.551 (16)	175
C4—H4*A*⋯F1^vi^	0.97	2.54	3.341 (19)	140
C3′—H3′*A*⋯O4^iii^	0.97	2.54	3.136 (17)	119
C4′—H4′*B*⋯O2	0.97	2.54	3.239 (14)	129

Symmetry codes: (i) 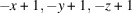; (ii) 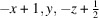; (iii) 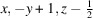; (iv) 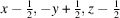; (v) 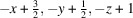; (vi) 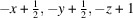.

**Table 3:** Experimental details

Crystal data	
Chemical formula	[Ni(C_24_H_36_F_2_N_6_)](ClO_4_)_2_
*M*_r_	704.20
Crystal system, space group	Monoclinic, *C*2/*c*
Temperature (K)	173
*a*, *b*, *c* (Å)	16.9910 (12), 15.5187 (11), 13.8864 (9)
β (°)	126.189 (1)
*V* (Å^3^)	2955.1 (4)
*Z*	4
Radiation type	Mo *K*α
μ (mm^−1^)	0.91
Crystal size (mm)	0.40 × 0.35 × 0.20

Data collection	
Diffractometer	Bruker SMART CCD area detector
No. of measured, independent and observed [*I* > 2σ(*I*)] reflections	9357, 3400, 2737
*R*_int_	0.083
(sin θ/λ)_max_ (Å^−1^)	0.667

Refinement	
*R*[*F* ^2^ > 2σ(*F* ^2^)], *wR*(*F* ^2^), *S*	0.068, 0.159, 1.13
No. of reflections	3400
No. of parameters	282
No. of restraints	492
H-atom treatment	H-atom parameters constrained
Δρ_max_, Δρ_min_ (e Å^−3^)	0.88, −0.63

Computer programs: *SMART* and *SAINT* (Bruker, 2002 ▸), *SHELXS97* (Sheldrick, 2008 ▸), *SHELXL2018/3* (Sheldrick, 2015 ▸), *ORTEP-3 for Windows* and *WinGX* (Farrugia, 2012 ▸), and *Mercury* (Macrae *et al.*, 2020 ▸).

## supplementary crystallographic information

### Crystal data

**Table:**

[Ni(C_24_H_36_F_2_N_6_)](ClO_4_)_2_	*F*(000) = 1464
*M**_r_* = 704.20	*D*_x_ = 1.583 Mg m^−^^3^
Monoclinic, *C*2/*c*	Mo *K*α radiation, λ = 0.71073 Å
*a* = 16.9910 (12) Å	Cell parameters from 3600 reflections
*b* = 15.5187 (11) Å	θ = 1.8–28.3°
*c* = 13.8864 (9) Å	µ = 0.91 mm^−^^1^
β = 126.189 (1)°	*T* = 173 K
*V* = 2955.1 (4) Å^3^	Block, yellow
*Z* = 4	0.40 × 0.35 × 0.20 mm

### Data collection

**Table:**

Bruker SMART CCD area detector diffractometer	*R*_int_ = 0.083
phi and ω scans	θ_max_ = 28.3°, θ_min_ = 2.0°
9357 measured reflections	*h* = −21→19
3400 independent reflections	*k* = −19→18
2737 reflections with *I* > 2σ(*I*)	*l* = −18→15

### Refinement

**Table:**

Refinement on *F*^2^	492 restraints
Least-squares matrix: full	Hydrogen site location: inferred from neighbouring sites
*R*[*F*^2^ > 2σ(*F*^2^)] = 0.068	H-atom parameters constrained
*wR*(*F*^2^) = 0.159	*w* = 1/[σ^2^(*F*_o_^2^) + (0.0449*P*)^2^ + 12.0119*P*] where *P* = (*F*_o_^2^ + 2*F*_c_^2^)/3
*S* = 1.13	(Δ/σ)_max_ < 0.001
3400 reflections	Δρ_max_ = 0.88 e Å^−^^3^
282 parameters	Δρ_min_ = −0.63 e Å^−^^3^

### Special details

**Table:**

Geometry. All esds (except the esd in the dihedral angle between two l.s. planes) are estimated using the full covariance matrix. The cell esds are taken into account individually in the estimation of esds in distances, angles and torsion angles; correlations between esds in cell parameters are only used when they are defined by crystal symmetry. An approximate (isotropic) treatment of cell esds is used for estimating esds involving l.s. planes.

### Fractional atomic coordinates and isotropic or equivalent isotropic displacement parameters (Å^2^)

**Table:**

	*x*	*y*	*z*	*U*_iso_*/*U*_eq_	Occ. (<1)
Ni1	0.50831 (10)	0.37625 (4)	0.25924 (13)	0.0174 (2)*	0.5
F1	0.0633 (8)	0.3851 (12)	0.5651 (8)	0.071 (3)	0.5
N1	0.2930 (6)	0.3576 (6)	0.2078 (8)	0.0457 (18)	0.5
N2	0.4195 (8)	0.4599 (6)	0.2494 (14)	0.0285 (16)	0.5
H2	0.440274	0.471036	0.330822	0.034*	0.5
N3	0.4422 (6)	0.2796 (5)	0.2689 (8)	0.0315 (17)	0.5
H3	0.464913	0.275912	0.352038	0.038*	0.5
C1	0.3129 (7)	0.4353 (7)	0.1751 (11)	0.0438 (19)	0.5
H1A	0.276898	0.481023	0.180846	0.053*	0.5
H1B	0.289042	0.431370	0.092254	0.053*	0.5
C2	0.3322 (7)	0.2838 (6)	0.1930 (9)	0.0407 (18)	0.5
H2A	0.307538	0.280024	0.109821	0.049*	0.5
H2B	0.308648	0.233687	0.210625	0.049*	0.5
C3	0.4317 (10)	0.5416 (7)	0.2027 (12)	0.043 (2)	0.5
H3A	0.398839	0.537629	0.117278	0.052*	0.5
H3B	0.404620	0.589837	0.218621	0.052*	0.5
C4	0.4734 (8)	0.1987 (5)	0.2428 (11)	0.042 (2)	0.5
H4A	0.462776	0.149576	0.277068	0.050*	0.5
H4B	0.436522	0.190069	0.157356	0.050*	0.5
C5	0.2772 (7)	0.3584 (7)	0.3011 (9)	0.043 (2)	0.5
H5A	0.324764	0.396071	0.365491	0.051*	0.5
H5B	0.286668	0.300782	0.333287	0.051*	0.5
C6	0.1754 (10)	0.3892 (15)	0.2521 (13)	0.0436 (17)	0.5
H6A	0.169116	0.448576	0.226476	0.052*	0.5
H6B	0.129079	0.355294	0.181870	0.052*	0.5
C7	0.1472 (8)	0.3846 (10)	0.3366 (10)	0.0368 (12)	0.5
C8	0.1429 (10)	0.3083 (10)	0.3863 (11)	0.048 (3)	0.5
H8	0.157393	0.256297	0.366611	0.057*	0.5
C9	0.1173 (10)	0.3085 (10)	0.4650 (11)	0.054 (3)	0.5
H9	0.119223	0.257617	0.501639	0.065*	0.5
C10	0.0891 (15)	0.3847 (12)	0.4885 (15)	0.0519 (18)	0.5
C11	0.0973 (19)	0.4610 (12)	0.447 (2)	0.046 (2)	0.5
H11	0.084266	0.512687	0.469261	0.055*	0.5
C12	0.126 (2)	0.4599 (11)	0.371 (2)	0.039 (2)	0.5
H12	0.130349	0.512186	0.342290	0.047*	0.5
F1'	0.9281 (8)	0.3557 (11)	−0.0646 (8)	0.071 (3)	0.5
N1'	0.7183 (6)	0.3953 (6)	0.3006 (8)	0.0457 (18)	0.5
N2'	0.5718 (8)	0.4730 (6)	0.2422 (15)	0.0315 (17)	0.5
H2'	0.548837	0.474605	0.158805	0.038*	0.5
N3'	0.5922 (6)	0.2927 (5)	0.2582 (8)	0.0285 (16)	0.5
H3'	0.570787	0.286557	0.175710	0.034*	0.5
C1'	0.6818 (7)	0.4693 (6)	0.3192 (10)	0.0407 (18)	0.5
H1'A	0.706114	0.519692	0.303050	0.049*	0.5
H1'B	0.706349	0.471549	0.402388	0.049*	0.5
C2'	0.6995 (7)	0.3171 (7)	0.3348 (10)	0.0438 (19)	0.5
H2'A	0.722435	0.321528	0.417275	0.053*	0.5
H2'B	0.736204	0.271471	0.330180	0.053*	0.5
C3'	0.5385 (10)	0.5534 (7)	0.2658 (12)	0.042 (2)	0.5
H3'A	0.551526	0.603098	0.234804	0.050*	0.5
H3'B	0.571198	0.561228	0.350715	0.050*	0.5
C4'	0.5786 (8)	0.2082 (5)	0.2969 (10)	0.043 (2)	0.5
H4'A	0.600078	0.161811	0.270542	0.052*	0.5
H4'B	0.616559	0.206027	0.383173	0.052*	0.5
C5'	0.7323 (7)	0.3938 (7)	0.2050 (9)	0.043 (2)	0.5
H5'A	0.695637	0.440331	0.149100	0.051*	0.5
H5'B	0.708406	0.339732	0.161773	0.051*	0.5
C6'	0.8384 (11)	0.4037 (15)	0.2587 (14)	0.0436 (17)	0.5
H6'A	0.874819	0.360440	0.320403	0.052*	0.5
H6'B	0.860033	0.459795	0.296500	0.052*	0.5
C7'	0.8622 (8)	0.3952 (10)	0.1701 (10)	0.0368 (12)	0.5
C8'	0.8807 (9)	0.3129 (9)	0.1490 (10)	0.039 (2)	0.5
H8'	0.875962	0.266112	0.187145	0.047*	0.5
C9'	0.9059 (10)	0.2994 (10)	0.0724 (11)	0.046 (2)	0.5
H9'	0.923553	0.245074	0.062804	0.055*	0.5
C10'	0.9039 (15)	0.3693 (11)	0.0113 (15)	0.0519 (18)	0.5
C11'	0.893 (2)	0.4515 (12)	0.034 (2)	0.054 (3)	0.5
H11'	0.902900	0.497780	−0.000650	0.065*	0.5
C12'	0.868 (2)	0.4641 (12)	0.112 (2)	0.048 (3)	0.5
H12'	0.854488	0.519427	0.124343	0.057*	0.5
Cl1	0.6115 (4)	0.3604 (3)	0.5699 (5)	0.0469 (4)	0.795 (7)
O1	0.6801 (6)	0.3549 (5)	0.6978 (5)	0.082 (3)	0.795 (7)
O2	0.5813 (5)	0.2776 (4)	0.5188 (5)	0.102 (2)	0.795 (7)
O3	0.6555 (5)	0.3981 (6)	0.5233 (6)	0.113 (3)	0.795 (7)
O4	0.5284 (5)	0.4091 (5)	0.5368 (6)	0.089 (2)	0.795 (7)
Cl1'	0.6086 (16)	0.3675 (13)	0.5680 (19)	0.0469 (4)	0.205 (7)
O1'	0.6497 (18)	0.3241 (14)	0.6781 (19)	0.049 (4)	0.205 (7)
O2'	0.6325 (17)	0.3288 (16)	0.4980 (16)	0.074 (4)	0.205 (7)
O3'	0.6267 (19)	0.4551 (13)	0.574 (2)	0.097 (6)	0.205 (7)
O4'	0.5058 (15)	0.3669 (16)	0.5057 (19)	0.067 (4)	0.205 (7)

### Atomic displacement parameters (Å^2^)

**Table:**

	*U*^11^	*U*^22^	*U*^33^	*U*^12^	*U*^13^	*U*^23^
F1	0.074 (3)	0.112 (11)	0.060 (2)	0.010 (4)	0.0571 (19)	0.004 (3)
N1	0.035 (2)	0.063 (6)	0.054 (3)	−0.001 (2)	0.034 (2)	0.005 (3)
N2	0.034 (3)	0.030 (3)	0.034 (3)	0.007 (2)	0.028 (3)	0.006 (3)
N3	0.037 (3)	0.031 (3)	0.039 (4)	−0.003 (3)	0.029 (3)	−0.002 (3)
C1	0.031 (3)	0.057 (5)	0.049 (4)	0.012 (3)	0.026 (3)	0.011 (4)
C2	0.038 (3)	0.050 (4)	0.043 (4)	−0.014 (3)	0.029 (3)	−0.007 (3)
C3	0.067 (6)	0.031 (3)	0.060 (6)	0.013 (3)	0.054 (5)	0.010 (3)
C4	0.073 (7)	0.022 (3)	0.062 (6)	−0.003 (3)	0.057 (6)	−0.001 (3)
C5	0.033 (3)	0.061 (8)	0.045 (3)	−0.001 (3)	0.028 (2)	0.002 (3)
C6	0.032 (4)	0.066 (6)	0.040 (3)	−0.003 (4)	0.025 (3)	−0.003 (5)
C7	0.029 (2)	0.052 (3)	0.033 (2)	0.003 (5)	0.020 (2)	−0.002 (4)
C8	0.045 (6)	0.053 (4)	0.046 (7)	0.009 (4)	0.027 (6)	0.010 (4)
C9	0.051 (7)	0.073 (5)	0.041 (7)	0.000 (5)	0.029 (6)	0.011 (5)
C10	0.041 (3)	0.086 (6)	0.041 (2)	0.010 (6)	0.031 (2)	0.008 (5)
C11	0.036 (5)	0.068 (4)	0.032 (5)	0.007 (4)	0.020 (5)	−0.003 (4)
C12	0.036 (5)	0.052 (4)	0.028 (5)	0.003 (4)	0.019 (4)	0.003 (4)
F1'	0.074 (3)	0.112 (11)	0.060 (2)	0.010 (4)	0.0571 (19)	0.004 (3)
N1'	0.035 (2)	0.063 (6)	0.054 (3)	−0.001 (2)	0.034 (2)	0.005 (3)
N2'	0.037 (3)	0.031 (3)	0.039 (4)	−0.003 (3)	0.029 (3)	−0.002 (3)
N3'	0.034 (3)	0.030 (3)	0.034 (3)	0.007 (2)	0.028 (3)	0.006 (3)
C1'	0.038 (3)	0.050 (4)	0.043 (4)	−0.014 (3)	0.029 (3)	−0.007 (3)
C2'	0.031 (3)	0.057 (5)	0.049 (4)	0.012 (3)	0.026 (3)	0.011 (4)
C3'	0.073 (7)	0.022 (3)	0.062 (6)	−0.003 (3)	0.057 (6)	−0.001 (3)
C4'	0.067 (6)	0.031 (3)	0.060 (6)	0.013 (3)	0.054 (5)	0.010 (3)
C5'	0.033 (3)	0.061 (8)	0.045 (3)	−0.001 (3)	0.028 (2)	0.002 (3)
C6'	0.032 (4)	0.066 (6)	0.040 (3)	−0.003 (4)	0.025 (3)	−0.003 (5)
C7'	0.029 (2)	0.052 (3)	0.033 (2)	0.003 (5)	0.020 (2)	−0.002 (4)
C8'	0.036 (5)	0.052 (4)	0.028 (5)	0.003 (4)	0.019 (4)	0.003 (4)
C9'	0.036 (5)	0.068 (4)	0.032 (5)	0.007 (4)	0.020 (5)	−0.003 (4)
C10'	0.041 (3)	0.086 (6)	0.041 (2)	0.010 (6)	0.031 (2)	0.008 (5)
C11'	0.051 (7)	0.073 (5)	0.041 (7)	0.000 (5)	0.029 (6)	0.011 (5)
C12'	0.045 (6)	0.053 (4)	0.046 (7)	0.009 (4)	0.027 (6)	0.010 (4)
Cl1	0.0583 (8)	0.0582 (11)	0.0380 (6)	0.0229 (7)	0.0361 (6)	0.0149 (7)
O1	0.089 (5)	0.116 (6)	0.038 (3)	0.058 (4)	0.035 (3)	0.014 (3)
O2	0.119 (5)	0.063 (3)	0.077 (4)	0.015 (3)	0.032 (3)	0.012 (3)
O3	0.113 (5)	0.162 (6)	0.094 (4)	−0.035 (4)	0.077 (4)	0.015 (4)
O4	0.081 (4)	0.108 (5)	0.062 (4)	0.056 (4)	0.033 (3)	0.010 (3)
Cl1'	0.0583 (8)	0.0582 (11)	0.0380 (6)	0.0229 (7)	0.0361 (6)	0.0149 (7)
O1'	0.068 (8)	0.052 (8)	0.050 (7)	0.028 (6)	0.047 (6)	0.017 (6)
O2'	0.093 (8)	0.095 (9)	0.039 (6)	0.040 (8)	0.042 (6)	0.016 (7)
O3'	0.108 (9)	0.081 (8)	0.084 (8)	0.002 (7)	0.046 (7)	0.013 (6)
O4'	0.073 (7)	0.093 (9)	0.050 (7)	0.007 (7)	0.044 (5)	0.008 (7)

### Geometric parameters (Å, º)

**Table:**

Ni1—N3	1.925 (7)	N1'—C1'	1.400 (11)
Ni1—N2	1.933 (9)	N1'—C2'	1.408 (12)
Ni1—N3'	1.934 (7)	N1'—C5'	1.481 (10)
Ni1—N2'	1.943 (9)	N2'—C3'	1.484 (10)
F1—C10	1.370 (10)	N2'—C1'	1.510 (11)
N1—C1	1.398 (11)	N2'—H2'	0.9800
N1—C2	1.401 (11)	N3'—C4'	1.486 (10)
N1—C5	1.469 (10)	N3'—C2'	1.519 (12)
N2—C3	1.495 (11)	N3'—H3'	0.9800
N2—C1	1.511 (11)	C1'—H1'A	0.9700
N2—H2	0.9800	C1'—H1'B	0.9700
N3—C4	1.488 (9)	C2'—H2'A	0.9700
N3—C2	1.511 (12)	C2'—H2'B	0.9700
N3—H3	0.9800	C3'—H3'A	0.9700
C1—H1A	0.9700	C3'—H3'B	0.9700
C1—H1B	0.9700	C4'—H4'A	0.9700
C2—H2A	0.9700	C4'—H4'B	0.9700
C2—H2B	0.9700	C5'—C6'	1.498 (12)
C3—C3'	1.489 (13)	C5'—H5'A	0.9700
C3—H3A	0.9700	C5'—H5'B	0.9700
C3—H3B	0.9700	C6'—C7'	1.510 (10)
C4—C4'	1.482 (17)	C6'—H6'A	0.9700
C4—H4A	0.9700	C6'—H6'B	0.9700
C4—H4B	0.9700	C7'—C12'	1.380 (11)
C5—C6	1.515 (11)	C7'—C8'	1.387 (11)
C5—H5A	0.9700	C8'—C9'	1.377 (11)
C5—H5B	0.9700	C8'—H8'	0.9300
C6—C7	1.509 (10)	C9'—C10'	1.366 (12)
C6—H6A	0.9700	C9'—H9'	0.9300
C6—H6B	0.9700	C10'—C11'	1.351 (12)
C7—C8	1.393 (11)	C11'—C12'	1.395 (12)
C7—C12	1.394 (11)	C11'—H11'	0.9300
C8—C9	1.394 (12)	C12'—H12'	0.9300
C8—H8	0.9300	Cl1—O3	1.376 (7)
C9—C10	1.385 (12)	Cl1—O2	1.410 (7)
C9—H9	0.9300	Cl1—O4	1.418 (6)
C10—C11	1.357 (12)	Cl1—O1	1.440 (6)
C11—C12	1.396 (11)	Cl1'—O3'	1.386 (17)
C11—H11	0.9300	Cl1'—O2'	1.392 (17)
C12—H12	0.9300	Cl1'—O4'	1.420 (17)
F1'—C10'	1.356 (10)	Cl1'—O1'	1.421 (16)
			
N2—Ni1—N3	93.7 (4)	C3'—N2'—C1'	110.4 (9)
N3—Ni1—N3'	86.6 (3)	C3'—N2'—Ni1	108.1 (8)
N2—Ni1—N3'	176.4 (4)	C1'—N2'—Ni1	115.4 (8)
N3—Ni1—N2'	177.5 (5)	C3'—N2'—H2'	107.5
N2—Ni1—N2'	86.5 (2)	C1'—N2'—H2'	107.5
N3'—Ni1—N2'	93.0 (4)	Ni1—N2'—H2'	107.5
C1—N1—C2	115.5 (7)	C4'—N3'—C2'	110.2 (8)
C1—N1—C5	118.9 (9)	C4'—N3'—Ni1	108.5 (6)
C2—N1—C5	119.1 (8)	C2'—N3'—Ni1	114.4 (6)
C3—N2—C1	109.6 (9)	C4'—N3'—H3'	107.9
C3—N2—Ni1	107.3 (7)	C2'—N3'—H3'	107.9
C1—N2—Ni1	116.7 (7)	Ni1—N3'—H3'	107.9
C3—N2—H2	107.6	N1'—C1'—N2'	113.4 (8)
C1—N2—H2	107.6	N1'—C1'—H1'A	108.9
Ni1—N2—H2	107.6	N2'—C1'—H1'A	108.9
C4—N3—C2	109.7 (7)	N1'—C1'—H1'B	108.9
C4—N3—Ni1	109.6 (5)	N2'—C1'—H1'B	108.9
C2—N3—Ni1	116.8 (6)	H1'A—C1'—H1'B	107.7
C4—N3—H3	106.7	N1'—C2'—N3'	113.3 (7)
C2—N3—H3	106.7	N1'—C2'—H2'A	108.9
Ni1—N3—H3	106.7	N3'—C2'—H2'A	108.9
N1—C1—N2	114.7 (8)	N1'—C2'—H2'B	108.9
N1—C1—H1A	108.6	N3'—C2'—H2'B	108.9
N2—C1—H1A	108.6	H2'A—C2'—H2'B	107.7
N1—C1—H1B	108.6	N2'—C3'—C3	105.0 (11)
N2—C1—H1B	108.6	N2'—C3'—H3'A	110.8
H1A—C1—H1B	107.6	C3—C3'—H3'A	110.8
N1—C2—N3	115.7 (7)	N2'—C3'—H3'B	110.8
N1—C2—H2A	108.4	C3—C3'—H3'B	110.8
N3—C2—H2A	108.4	H3'A—C3'—H3'B	108.8
N1—C2—H2B	108.4	C4—C4'—N3'	107.6 (8)
N3—C2—H2B	108.4	C4—C4'—H4'A	110.2
H2A—C2—H2B	107.4	N3'—C4'—H4'A	110.2
C3'—C3—N2	106.4 (10)	C4—C4'—H4'B	110.2
C3'—C3—H3A	110.4	N3'—C4'—H4'B	110.2
N2—C3—H3A	110.4	H4'A—C4'—H4'B	108.5
C3'—C3—H3B	110.4	N1'—C5'—C6'	109.6 (8)
N2—C3—H3B	110.4	N1'—C5'—H5'A	109.8
H3A—C3—H3B	108.6	C6'—C5'—H5'A	109.8
C4'—C4—N3	106.8 (8)	N1'—C5'—H5'B	109.8
C4'—C4—H4A	110.4	C6'—C5'—H5'B	109.8
N3—C4—H4A	110.4	H5'A—C5'—H5'B	108.2
C4'—C4—H4B	110.4	C5'—C6'—C7'	114.0 (8)
N3—C4—H4B	110.4	C5'—C6'—H6'A	108.7
H4A—C4—H4B	108.6	C7'—C6'—H6'A	108.7
N1—C5—C6	111.1 (8)	C5'—C6'—H6'B	108.7
N1—C5—H5A	109.4	C7'—C6'—H6'B	108.7
C6—C5—H5A	109.4	H6'A—C6'—H6'B	107.6
N1—C5—H5B	109.4	C12'—C7'—C8'	119.1 (9)
C6—C5—H5B	109.4	C12'—C7'—C6'	123.8 (11)
H5A—C5—H5B	108.0	C8'—C7'—C6'	117.1 (11)
C7—C6—C5	116.0 (8)	C9'—C8'—C7'	121.1 (10)
C7—C6—H6A	108.3	C9'—C8'—H8'	119.4
C5—C6—H6A	108.3	C7'—C8'—H8'	119.4
C7—C6—H6B	108.3	C10'—C9'—C8'	117.0 (10)
C5—C6—H6B	108.3	C10'—C9'—H9'	121.5
H6A—C6—H6B	107.4	C8'—C9'—H9'	121.5
C8—C7—C12	116.1 (9)	C11'—C10'—F1'	118.1 (11)
C8—C7—C6	124.0 (11)	C11'—C10'—C9'	124.3 (10)
C12—C7—C6	119.9 (11)	F1'—C10'—C9'	116.8 (12)
C7—C8—C9	121.1 (11)	C10'—C11'—C12'	117.2 (13)
C7—C8—H8	119.4	C10'—C11'—H11'	121.4
C9—C8—H8	119.4	C12'—C11'—H11'	121.4
C10—C9—C8	119.9 (11)	C7'—C12'—C11'	120.5 (13)
C10—C9—H9	120.0	C7'—C12'—H12'	119.8
C8—C9—H9	120.0	C11'—C12'—H12'	119.8
C11—C10—F1	118.8 (12)	O3—Cl1—O2	106.7 (6)
C11—C10—C9	120.7 (10)	O3—Cl1—O4	109.5 (6)
F1—C10—C9	120.1 (11)	O2—Cl1—O4	109.0 (6)
C10—C11—C12	118.4 (12)	O3—Cl1—O1	109.6 (6)
C10—C11—H11	120.8	O2—Cl1—O1	110.9 (5)
C12—C11—H11	120.8	O4—Cl1—O1	110.9 (5)
C7—C12—C11	123.4 (12)	O3'—Cl1'—O2'	108.4 (19)
C7—C12—H12	118.3	O3'—Cl1'—O4'	101.1 (17)
C11—C12—H12	118.3	O2'—Cl1'—O4'	109.5 (18)
C1'—N1'—C2'	115.6 (7)	O3'—Cl1'—O1'	116.9 (18)
C1'—N1'—C5'	120.1 (8)	O2'—Cl1'—O1'	112.8 (18)
C2'—N1'—C5'	118.5 (9)	O4'—Cl1'—O1'	107.4 (18)
			
C2—N1—C1—N2	−64.3 (13)	C5'—N1'—C1'—N2'	86.6 (11)
C5—N1—C1—N2	87.3 (12)	C3'—N2'—C1'—N1'	−179.4 (10)
C3—N2—C1—N1	177.6 (10)	Ni1—N2'—C1'—N1'	57.6 (13)
Ni1—N2—C1—N1	55.4 (13)	C1'—N1'—C2'—N3'	67.6 (12)
C1—N1—C2—N3	63.7 (12)	C5'—N1'—C2'—N3'	−85.8 (10)
C5—N1—C2—N3	−87.7 (11)	C4'—N3'—C2'—N1'	178.0 (8)
C4—N3—C2—N1	−179.3 (8)	Ni1—N3'—C2'—N1'	−59.5 (10)
Ni1—N3—C2—N1	−53.9 (10)	C1'—N2'—C3'—C3	−169.8 (8)
C1—N2—C3—C3'	−170.4 (8)	Ni1—N2'—C3'—C3	−42.7 (11)
Ni1—N2—C3—C3'	−42.7 (11)	N2—C3—C3'—N2'	55.7 (7)
C2—N3—C4—C4'	166.7 (7)	N3—C4—C4'—N3'	−49.6 (8)
Ni1—N3—C4—C4'	37.3 (9)	C2'—N3'—C4'—C4	165.1 (7)
C1—N1—C5—C6	78.6 (14)	Ni1—N3'—C4'—C4	39.3 (9)
C2—N1—C5—C6	−130.9 (14)	C1'—N1'—C5'—C6'	103.5 (13)
N1—C5—C6—C7	174.2 (12)	C2'—N1'—C5'—C6'	−104.4 (13)
C5—C6—C7—C8	−61.5 (16)	N1'—C5'—C6'—C7'	174.9 (12)
C5—C6—C7—C12	117 (2)	C5'—C6'—C7'—C12'	93 (2)
C12—C7—C8—C9	0.7 (16)	C5'—C6'—C7'—C8'	−88.0 (15)
C6—C7—C8—C9	179.3 (10)	C12'—C7'—C8'—C9'	1.0 (16)
C7—C8—C9—C10	4 (2)	C6'—C7'—C8'—C9'	−178.0 (10)
C8—C9—C10—C11	−8 (2)	C7'—C8'—C9'—C10'	−5.3 (19)
C8—C9—C10—F1	179.6 (14)	C8'—C9'—C10'—C11'	10 (3)
F1—C10—C11—C12	179 (2)	C8'—C9'—C10'—F1'	−179.6 (13)
C9—C10—C11—C12	6 (3)	F1'—C10'—C11'—C12'	180 (2)
C8—C7—C12—C11	−3 (3)	C9'—C10'—C11'—C12'	−11 (3)
C6—C7—C12—C11	178.8 (18)	C8'—C7'—C12'—C11'	−1 (3)
C10—C11—C12—C7	−1 (4)	C6'—C7'—C12'—C11'	177.8 (18)
C2'—N1'—C1'—N2'	−66.3 (12)	C10'—C11'—C12'—C7'	6 (4)

### Hydrogen-bond geometry (Å, º)

**Table:**

*D*—H···*A*	*D*—H	H···*A*	*D*···*A*	*D*—H···*A*
N2—H2···O4	0.98	2.51	3.355 (17)	144
N2—H2···O4^i^	0.98	2.44	3.260 (16)	141
N2′—H2′···O4^ii^	0.98	2.43	3.332 (18)	152
N2′—H2′···O4^iii^	0.98	2.36	3.089 (17)	131
N3—H3···O2	0.98	1.97	2.819 (11)	144
N3′—H3′···O2^ii^	0.98	2.40	3.186 (11)	137
C1′—H1′*A*···O1^iii^	0.97	2.31	3.198 (13)	151
C1—H1*B*···O3^ii^	0.97	2.35	3.156 (16)	140
C1′—H1′*B*···O3	0.97	2.56	3.309 (15)	134
C2—H2*B*···O1^iv^	0.97	2.50	3.394 (16)	154
C3—H3*B*···O1^i^	0.97	2.48	3.35 (2)	149
C2′—H2′*B*···O1^v^	0.97	2.58	3.551 (16)	175
C4—H4*A*···F1^vi^	0.97	2.54	3.341 (19)	140
C3′—H3′*A*···O4^iii^	0.97	2.54	3.136 (17)	119
C4′—H4′*B*···O2	0.97	2.54	3.239 (14)	129

Symmetry codes: (i) −*x*+1, −*y*+1, −*z*+1; (ii) −*x*+1, *y*, −*z*+1/2; (iii) *x*, −*y*+1, *z*−1/2; (iv) *x*−1/2, −*y*+1/2, *z*−1/2; (v) −*x*+3/2, −*y*+1/2, −*z*+1; (vi) −*x*+1/2, −*y*+1/2, −*z*+1.
